# Application of magnetic resonance image compilation (MAGiC) in the diagnosis of middle-aged and elderly women with osteoporosis

**DOI:** 10.1186/s12880-023-01010-9

**Published:** 2023-05-15

**Authors:** Yiming Chen, Xiuting Mei, Xuqian Liang, Yi Cao, Cong Peng, Yang Fu, Yulong Zhang, Cuifang Liu, Yang Liu

**Affiliations:** 1 Radiology Department of Chongqing Traditional Chinese Medicine Hospital, Chongqing, China; 2 Rehabilitation Department of Chongqing Traditional Chinese Medicine Hospital, Chongqing, China; 3 School of Pharmacy, Chongqing Medical and Pharmaceutical College, Chongqing, China

**Keywords:** Osteoporosis, Magnetic resonance imaging, Magnetic resonance image compilation, Relaxometry, Ridge regression

## Abstract

**Objective:**

To investigate the feasibility of diagnosing osteoporosis (OP) in women through magnetic resonance image compilation (MAGiC).

**Methods:**

A total of 110 patients who underwent lumbar magnetic resonance imaging and dual X-ray absorptiometry examinations were collected and divided into two groups according bone mineral density: osteoporotic group (OP) and non-osteoporotic group (non-OP). The variation trends of T1 (longitudinal relaxation time), T2 (transverse relaxation time) and BMD (bone mineral density) with the increase of age, and the correlation of T1 and T2 with BMD were examined by establishing a clinical mathematical model.

**Results:**

With the increase of age, BMD and T1 value decreased gradually, while T2 value increased. T1 and T2 had statistical significance in diagnosing OP (*P < 0.001*), and there is moderate positive correlation between T1 and BMD values (R = 0.636, *P < 0.001*), while moderate negative correlation between T2 and BMD values (R=-0.694, *P < 0.001*). Receiver characteristic curve test showed that T1 and T2 had high accuracy in diagnosing OP (T1 AUC = 0.982, T2 AUC = 0.978), and the critical values of T1 and T2 for evaluating osteoporosis were 0.625s and 0.095s, respectively. Besides, the combined utilization of T1 and T2 had higher diagnostic efficiency (AUC = 0.985). Combined T1 and T2 had higher diagnostic efficiency (AUC = 0.985). Function fitting results of OP group: BMD=-0.0037* age − 0.0015*T1 + 0.0037*T2 + 0.86, sum of squared error (SSE) = 0.0392, and non-OP group: BMD = 0.0024* age − 0.0071*T1 + 0.0007*T2 + 1.41, SSE = 0.1007.

**Conclusion:**

T1 and T2 value of MAGiC have high efficiency in diagnosing OP by establishing a function fitting formula of BMD with T1, T2 and age.

## Introduction

Osteoporosis (OP) is a common systemic metabolic disease characterized by decreased bone mineral density (BMD), structural deterioration of bone tissue, and increased fat fraction (FF) in bone marrow, which leads to bone fragility and increased risk of bone fracture [1, 2]. The incidence of OP in China has ranked first in the world, and its prevalence increases with age [3]. OP has become one of the most common causes of fracture in the middle-aged and elderly, especially in women, bringing a heavy burden to the family and society. In order to assess bone quality and diagnose OP or osteopenia in its early phase, radiologic assessments are often ordered for making decisions regarding the appropriate therapeutic measures. Currently, the radiologic diagnosis of OP mainly includes dual X-ray absorptiometry (DXA) [4], quantitative computed tomography (QCT) [5, 6], magnetic resonance imaging (MRI), and nuclear medicine. In consideration of radiation damage of DXA and QCT, advanced MRI technologies are more and more applied on the early diagnosis of OP in recent studies [7–9].

MRI has the advantages of radiation free and high soft tissue contrast. Compared with X-ray and CT, early changes of bone marrow such as edema can be sensitively detected by MRI. In addition, MRI has unique advantages in displaying subtle fractures, and distinguishing bone tumor and infection. Direct measurement of conventional MRI signal intensity alone cannot reflect the characteristic changes in composition of the bone marrow. Recent studies of the evaluation of bone marrow fat content by MRI fat measurement sequence suggested that OP could be evaluated by conventional lipid suppression technology of short TI inversion recovery (STIR), iterative decomposition of water and fat with echo asymmetry and least-squares (IDEAL), diffusion weighted imaging (DWI) and magnetic resonance spectroscopy (MRS), etc. However, due to small clinical study population, and lack of correlations between MRI quantitative parameters and BMD, the above techniques fail to provide direct BMD values and clinical diagnosis of OP. Magnetic resonance image compilation (MAGiC) is a new quantitative scanning technology based on the principle of multiple-delay multiple-echo (MDME) sequences. In the meantime, four 120° saturation pulses were applied in different TR time and double echo acquisition was carried out. Ten contrast weighted images and five quantitative relaxation images were obtained by combining two TE and four TI at one time. In addition to obtaining conventional contrast images, MAGiC can also be applied to reconstruct phase sensitive inversion recover (PSIR), double inversion recovery-white matter or Grey matter (DIR-WM/GM), which provides high diagnostic value for multiple sclerosis [10–12], facial hemangioma syndrome [13, 14] and other lesions.

MAGiC can quantitatively measure intrinsic properties of tissues based on longitudinal relaxation time (T1), transverse relaxation time (T2) and proton density (PD) parameters. Accurate quantification of T1 and T2 values is important in quantitative MRI technology. Although the relaxation characteristics of tissue are basically stable under the condition of fixed magnetic field, interference within sequences is often noticed in weighted images obtained from conventional MRI sequences. Therefore, direct measurement of the signal intensity value doesn’t reflect the actual characteristics of tissues. In contrast, quantitative MRI technology such as MAGiC sequence simultaneously obtain quantitative maps of T1 and T2 to achieve quantification of tissue characteristics, and changes in T1 and T2 values are related to tissue changes, providing a basis for morphological diagnosis to quantitative diagnosis [15].

Currently, MRI MAGiC sequence is mostly clinically applied in the diagnosis of soft tissue diseases, while few studies investigated the potential of MRI MAGiC sequence in musculoskeletal disorder, especially OP diagnosis [16–19]. The present study aims to provide a safe and effective strategy for OP diagnosis and screening by comparing and analyzing the trend of MAGiC quantitative parameters and BMD in middle-aged and elderly women.

## Materials and methods

### Subject population

One-hundred and ten female patients (53.01 ± 16.99 years old, ranged from 26 to 91 years old) underwent lumbar MAGiC examination and DXA scan in Chongqing Hospital of Traditional Chinese Medicine from November 2020 to April 2021 were enrolled. All subjects were excluded from history of violent traumatic fractures, lumbar surgery, tumors, metabolic diseases and blood diseases, taking hormones, and immune rheumatic diseases. According to DXA diagnosis results, the patients were divided into OP case group (56 cases, all ≥ 50 years old) and non-OP control group (54 cases, all < 50 years old), different from age-standardized prevalence (60 years old) of OP in Chinese women [4]. No obvious compression changes in lumbar body height and STIR sequence high signal signs were noticed during MAGiC examination. This study was approved by Ethics Committee of Chongqing Traditional Chinese Medicine Hospital (2021-ky-43), carried out in accordance with The Code of Ethics of the World Medical Association (Declaration of Helsinki) for experiments involving humans, and informed consents were obtained from all patients.

### MR and DXA examinations

Lumbar magnetic resonance was examined by General Electric Company (GE) 3.0T SIGNA Architect MRI scanner with a 40-channel phased array spinal coil. To match the position of DXA lumbar scan, coronal FSE-T1WI, T2WI and MAGiC sequences (Table 1) were added in addition to conventional sagittal FSE-T1WI, FRFSE-T2WI, T2WI-Special and transverse FRFSE-T2WI Prop sequences. FOV, layer thickness and layer spacing (4 mm and 1 mm) were kept consistent in all sequences.

**Table 1:** Comparison in parameters of routine and MAGiC sequences for lumbar MRI

Sequence	TR ( ms )	TE ( ms )	Matrix	ETL	Bandwidth ( ms )	TA
T1WI-FSE	662	42	320 × 288	4	62.5	1min20s
FRFSE-T2WI	2589	102	320 × 288	2	62.5	1min8s
T2WI-special	3611	68	320 × 288	18	62.5	1min34s
Axi T2FRFSE Prop	3811	32	288 × 288	32	62.5	1min51s
Cor-FSE T1WI	662	42	320 × 288	4	62.5	1min20s
MAGiC	4000	14.4/86.5	320 × 256	16	50	4min0s

DXA bone mineral density BMD examination was conducted with discovery-WI dual-energy X-ray bone mineral density instrument (HOLOGIC, Inc., USA). During the examination, anteroposterior 2–4 vertebrae with no severe hyperostosis region were selected and analyzed.

### Quantitative analysis

Lumbar MAGiC images were double-blind processed by three experienced doctors (with Orthopedics related imaging diagnosis experience for over 15 years). The maximum range of cancellous bone of lumbar 2–4 vertebral body was manually selected for measurement. The bone cortex and intervertebral disc were avoided. The mean value and standard deviation of T1 and T2 values of lumbar 2–4 vertebral bodies were calculated and compared with BMD value of DXA examination. The function fitting formula of BMD (g/cm^2^) with T1 (s), T2 (s) and age was established by using Python program and Ridge regression model, sum of squared error (SSE) was used to evaluate fitting precision.

### Statistical analysis

Statistical analyses were performed using SPSS statistical software (version 18.0). Age-distributed lumbar relaxation time (T1, T2) and BMD results, as well as the grouped data of lumbar T1 and T2 were analyzed with single sample Kolmogorov-Smirnov test in OP and control groups. Paired T test was used for T1, T2 and BMD for parametric data, Mann-Whitney U test was used for nonparametric data. For the correlation statistical analysis between T1/T2 and BMD, as they follow Gaussian distribution, the Pearson product-moment correlation coefficient (PPMCC, R) was used, |R| < 0.4 was denoted as low correlation, 0.4 ≤ |R| ≤ 0.7 was denoted as moderate correlation and |R| > 0.7 was denoted as high correlation. Receiver Operating Characteristic (ROC) of subjects was drawn with Medcalc statistical software (version 12.7.7), and the efficiency difference of T1 and T2 in OP detection was compared and analyzed. The Logistic regression analysis was combined with the comprehensive diagnostic efficiency of T1 and T2. The maximum area under the curve (AUC) and the optimum Youden index were calculated. *P* < 0.05 was considered statistically significant.

## Results

**Fig. 1:**
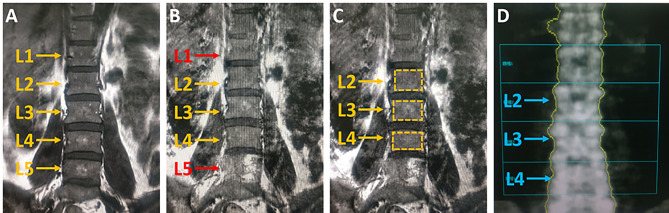
Schematic diagram T1 relaxation time measurement. (A) A conventional T1WI image with few artifacts, high signal-to-noise ratio, and good display of L1-5 vertebral structure and signal characteristics. (B) MAGiC T1 image showed poor display of L1 and L5 vertebrae (indicated with red arrows), mainly due to heavy respiratory motion artifacts. Thus, L2-4 vertebrae were selected for measurements. (C) The maximum ROI included the cancellous bone of the entire vertebrae, avoided the bone cortex and intervertebral disc as indicated with yellow rectangles. (D) The image of L2-4 BMD measured by bone density meter

**Fig. 2:**
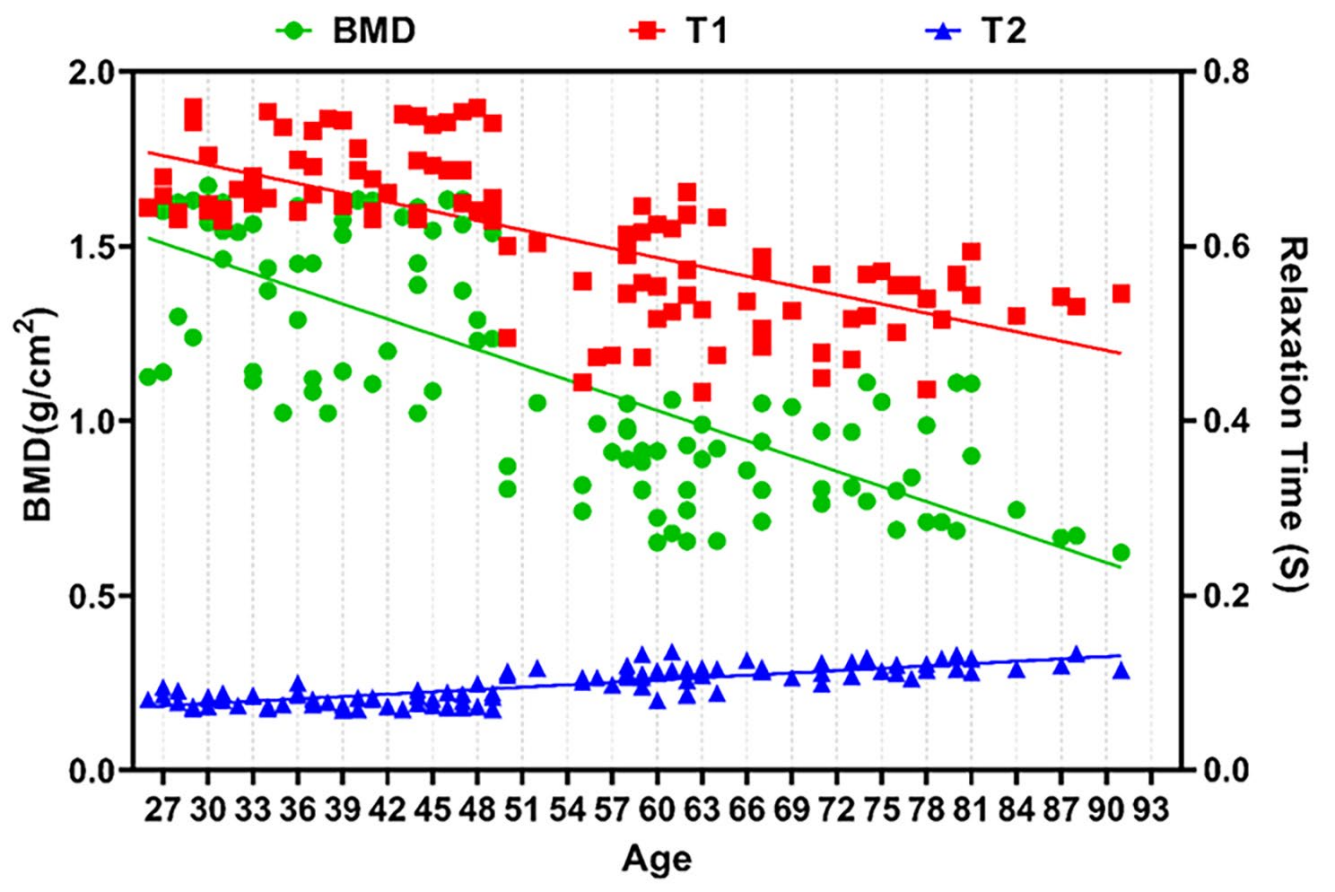
The variation trends of lumbar BMD, T1 and T2 values in middle-aged and elderly women with OP.

Typical measurement of T1 and T2 values for 2–4 vertebral bodies were shown in Fig. 1. The mean relaxation time of lumbar 2–4 vertebral body in all participants were T1 = 0.612 ± 0.085 s, T2 = 0.097 ± 0.019 s, and BMD = 1.131 ± 0.333 g/cm^2^. With the increase of age, lumbar BMD gradually decreased (Fig. 2). The T1 value was consistent with BMD change, presenting a gradual downward trend (R_bmd_=-0.742, *P < 0.001*;R_T1_=-0.706, *P < 0.001*), while the T2 value slightly increased (R_T2_=0.797, *P < 0.001*) (Fig. 2). Moderate positive correlation between T1 and BMD values (R = 0.636, *P < 0.001*), moderate negative correlation between T2 and BMD value (R=-0.694, P < 0.001), and negative correlation between T1 and T2 values (R=-0.781, P < 0.001) were noticed.

According to the results of DXA diagnosis, the 110 participants were divided into OP group (56 cases) and non-OP group (54 cases). The independent sample T test (Table 2) indicated statistical significances of relaxation time (T1 and T2) between OP and non-OP groups.

**Table 2:** t-test results of OP group and non-OP group

Group	OP	None-OP	F	*P*
T1 (s)	0.544 ± 0.557	0.681 ± 0.440	1.304	0.000
T2 (s)	0.112 ± 0.011	0.080 ± 0.007	3.429	0.000

**Fig. 3:**
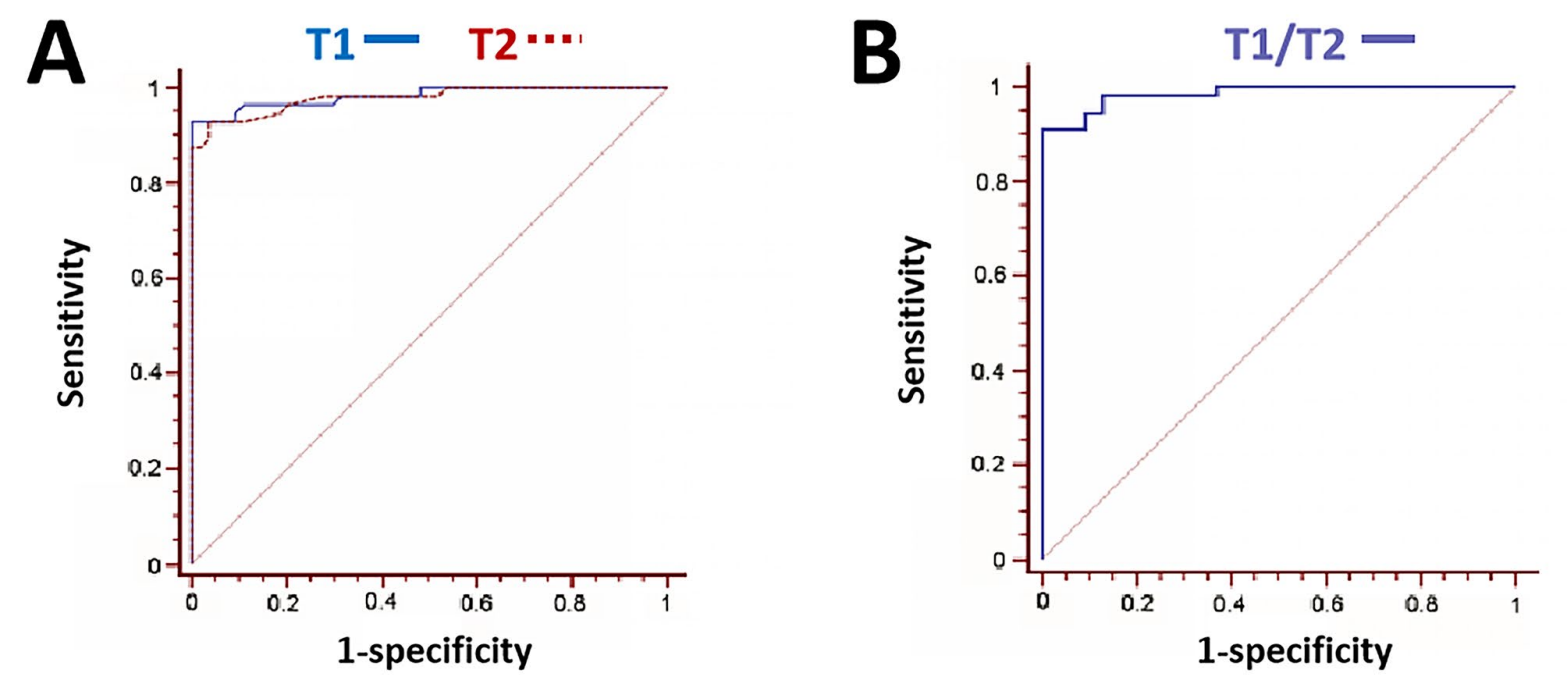
ROC curves of T1 and T2 for OP detection. (A) Comparison of T1 and T2 OP diagnostic. (B) T1 and T2 combined diagnosis showed higher accuracy than single parameter

As shown in Fig. 3, the maximum area under the receiver characteristic (ROC) curves suggested that T1 and T2 values of the lumbar spine effectively reflected the occurrence of OP. No significant difference in the diagnostic accuracy of OP between T1 and T2 measurements (*P* > 0.05) were noticed. In addition, the accuracy of combined detection of OP with T1 and T2 values was higher than that of T1 or T2 alone.

As shown in Table 3, the optimal cut off points of T1 and T2 values were 0.625 s and 0.095 s, respectively. Function fitting results using Matlab were as follow: OP group, BMD=-0.0037×age-0.0015×T1 + 0.0037×T2 + 0.86, SSE = 0.0392, Non-OP group, BMD = 0.0024×age•0.0071×T1 + 0.0007×T2 + 1.41, SSE = 0.1007. As suggested by SSE values, we considered that the function fitted by OP group is closer to the actual situation.

**Table 3:** ROC test results of T1 and T2 in diagnosing OP

	AUC (95%)	Z Value	*P*	Youden Index	Cutoff Point
T1	0.982	44.86	< 0.0001	0.9286	≤ 0.625
T2	0.978	40.668	< 0.0001	0.8915	> 0.095
T1-T2 difference		0.337	0.7364		
T1-T2 combine	0.985	57.794	< 0.0001	0.9107	

## Discussion

OP often occurs in middle-aged and postmenopausal women, which is mainly characterized by decreased bone mass and increased bone fragility. A large sample epidemiological survey showed that the number of female patients with OP (49.3 million) was significantly higher than that of male patients (10.9 million) aged 50 and above in China [20], indicating age and gender were the main influencing factors of OP. Early diagnosis in the asymptomatic stage is particularly important in middle-aged and women population. Currently, the diagnosis, risk prediction and efficacy evaluation of OP are mainly dependent on X-ray, CT, MRI, nuclear medicine, DXA, and QCT. Among these techniques, MRI has attracted more and more attention in OP research due to its non-radiation property, high tissue contrast, and sensitivity to early changes in bone tissue. However, conventional MRI signals are often interfered, and lack of unified quantitative standard. Direct measurement of signal intensity itself provides little information on tissue structural changes.

Currently, most MR techniques for quantitative evaluation of OP mainly focus on fat content, including spectral imaging, water-lipid separation imaging, and magnetic resonance fat suppression [21–24]. However, certain controversies exist in the detection results of quantitative fat evaluation of OP due to the uneven distribution of transformed yellow bone marrow, and other uncertainty of changes in the proportion of bone component [25, 26]. Yeung et al. [27] reported that vertebral body fat fraction in OP group was significantly higher than that in normal BMD population, mainly due to the increase of saturated fatty acids, and BMD was positively correlated with unsaturated fatty acids. However, Griffith et al. [26] found that there was no significant difference in the composition and content of bone marrow fatty acids in different BMD groups, and the relative content remained unchanged with age. On this basis, there is an urgent need for a new quantitative magnetic resonance technology to reflect structural alternation in bone tissue for OP diagnosis.

As a novel MR quantitative technology, MAGiC quantifies the relaxation time and proton density through multi-echo acquisition saturation recovery, and automatically reconstructs contrast and quantitative relaxation rate maps through post-processing. In addition, MAGiC is able to reconstruct tissue contrast images of any required TR, TE and TI parameters through post-parameter setting. During the scanning process, the small rotation pulses used in the previous RF excitation are replaced with 90° pulses and a series of 180° reunion pulses, which are combined to generate a series of spin echoes rather than gradient echoes to avoid magnetic field inhomogeneity and magneto-sensitive effect. Previous study [28] suggested that the overall quality, anatomical morphology, and ability of lesion detection in MAGiC images were comparable with those of conventional sequences. However, in this study, although voluntary movement was absence in the process of inspection, and the respiratory motion artifact was attenuated by saturated zone, and the reconstruction of the MAGiC figure displayed impaired signal-to-noise ratio at L1 and L5 vertebral bodies. Even though the image quality of MAGiC is not ideal, the efficacy for disease diagnosis of MAGiC has been verified by multi-center and repeatability studies [28–30]. In addition, compared with traditional T1 and T2 acquisition, MAGiC achieves T1 and T2 acquisition at the same time, with a much shorter scanning time, which might reduce motion artifacts, and increase patient cooperation during examination [13].

In an early study, Dooms et al. declared that T1 and T2 value were both negatively correlated with age, as the fat content increased and water content decreased in the vertebra [31]. However, they also mentioned that in post-menopause OP, the weakened magnetization effect at the interface between bone marrow and bone trabeculae could prolong T2 relaxation time. Now, T1 and T2 values were considered to lay different emphasis on spinal anatomy. Standardized measurement of the spine showed that T1 value was more affected by vertebral body alternation, while T2 value was correlated with structural changes in spinal cord and intervertebral disc [11]. Teng Hsiang-Ling et al. demonstrated that T2 value was more sensitive and positively correlated to the water content of bone tissue [32]. In the current study, lumbar T1 value was negatively correlated with age (R=-0.706, *P < 0.001*), which might be explained by the transformation of bone marrow from red to yellow in vertebral body [33, 34]. Since bone marrow transformation in vertebrae is focal and inhomogeneity [14], we outlined the maximum range of vertebrae during measurement to increase the accuracy of measurement. In contrast to T1 value, T2 value of the vertebral body had the same trend with age (R = 0.797, *P < 0.001*), suggesting increased bone absorption and free water (long T2) that theoretically increased in OP bone trabecula.

The accuracy of the two values in the diagnosis of OP was relatively high: T1 sensitivity was 92.86% with 100% specificity, T2 sensitivity was 92.86% with 96.30% specificity, suggesting that when T1 ≤ 0.625s or T2 > 0.095s in lumbar spine of women over 50 years old, the MRI findings are consistent with OP manifestations. However, it is important to notice that the overall accuracy (AUC 0.985) of combined T1 and T2 in diagnosing OP was higher than that of T1 (AUC 0.982) or T2 (AUC 0.978) alone, while the sensitivity (91.07%) was lower. Multiple linear regression might be necessary to screen optimal parameters for OP diagnosis. To obtain the function formula between OP and none-OP groups, ridge regression with more practical and reliable regression coefficients in solving the problem of collinearity of independent variables in linear regression analysis was applied. According to the formula, the non-op value of SSE_OP_ was smaller than that of SSEnon-op, and the range of verified data was 5%. It was considered that the function fitted by OP group was closer to the actual situation, and the corresponding BMD value could be directly calculated by female age and measurement of T1 and T2. Since traditional diagnosis of OP by DXA might not reflect the biomechanical properties of bone such as strength and viscoelasticity, which are closely related to bone water and organic matter and contribute to the mechanical strength of bone tissue [15]. MAGiC sequence might compensate these shortcomings of traditional detection.

## Conclusions

The current study provided a safe and effective strategy for OP diagnosis and screening by comparing and analyzing the trend of MAGiC quantitative parameters (T1 and T2) and BMD in middle-aged and elderly women, suggesting its potential application in early diagnosis of OP.

## Data Availability

The datasets used and/or analysed during the current study are available from the corresponding author on reasonable request.

## Abbreviations

OP: osteoporosis
MAGiC: magnetic resonance image compilation
T1: longitudinal relaxation time
T2: transverse relaxation time
BMD: bone mineral density
FF: fat fraction
DXA: dual X-ray absorptiometry
QCT: quantitative computed tomography
MRI: magnetic resonance imaging
STIR: short TI inversion recovery
IDEAL: iterative decomposition of water and fat with echo asymmetry and least-squares
DWI: diffusion weighted imaging
MRS: magnetic resonance spectroscopy
MDME: multiple-delay multiple-echo
PSIR: phase sensitive inversion recover
DIR-WM/GM: double inversion recovery-white matter or Grey matter
PD: proton density
ROC: Receiver Operating Characteristic
AUC: area under the curve.
